# Virus genome dynamics under different propagation pressures: reconstruction of whole genome haplotypes of west nile viruses from NGS data

**DOI:** 10.1186/s12864-015-1340-8

**Published:** 2015-02-22

**Authors:** Cornell Kortenhoeven, Fourie Joubert, Armanda DS Bastos, Celia Abolnik

**Affiliations:** Poultry Section, Department of Production Animal Studies, Faculty of Veterinary Science, University of Pretoria, Old Soutpan Road, Onderstepoort, 0110 South Africa; Department of Zoology and Entomology, Faculty of Natural and Agricultural Sciences, Mammal Research Institute, University of Pretoria, Lynwood Road, Pretoria, South Africa; ARC-Ondestepoort Veterinary Institute, 100 Old Soutpan Road, Onderstepoort, 0110 South Africa; Department of Biochemistry, Faculty of Natural and Agricultural Sciences, University of Pretoria, Lynwood Road, Pretoria, South Africa

**Keywords:** West Nile virus, Quasispecies Reconstruction, Mutation-Selection Equilibrium, Cell Tropism

## Abstract

**Background:**

Extensive focus is placed on the comparative analyses of consensus genotypes in the study of West Nile virus (WNV) emergence. Few studies account for genetic change in the underlying WNV quasispecies population variants. These variants are not discernable in the consensus genome at the time of emergence, and the maintenance of mutation-selection equilibria of population variants is greatly underestimated. The emergence of lineage 1 WNV strains has been studied extensively, but recent epidemics caused by lineage 2 WNV strains in Hungary, Austria, Greece and Italy emphasizes the increasing importance of this lineage to public health. In this study we explored the quasispecies dynamics of minority variants that contribute to cell-tropism and host determination, i.e. the ability to infect different cell types or cells from different species from Next Generation Sequencing (NGS) data of a historic lineage 2 WNV strain.

**Results:**

Minority variants contributing to host cell membrane association persist in the viral population without contributing to the genetic change in the consensus genome. Minority variants are shown to maintain a stable mutation-selection equilibrium under positive selection, particularly in the capsid gene region.

**Conclusions:**

This study is the first to infer positive selection and the persistence of WNV haplotype variants that contribute to viral fitness without accompanying genetic change in the consensus genotype, documented solely from NGS sequence data. The approach used in this study streamlines the experimental design seeking viral minority variants accurately from NGS data whilst minimizing the influence of associated sequence error.

## Background

The increase in outbreaks of severe and fatal neurological disease caused by West Nile Virus (WNV; *Flaviridae; Flavivirus*) is a cause for concern worldwide. This emerging zoonotic pathogen is primarily transmitted by *Culex* species mosquitoes in enzootic cycles where migratory birds serve as the reservoir host [1]. The frequency of infections in incidental hosts, including humans and horses has increased over the past decade [2].

WNV has a positive-sense single-stranded RNA genome approximately 11 Kb in length [1]. The WNV genome encodes a single open reading frame (ORF) which is flanked by 5′ and 3′ untranslated regions (UTRs) [3]. The polyprotein, approximately 3000 amino acid (aa) in length, is cleaved into three structural proteins and seven non-structural proteins [3]. The structural proteins are required for virion formation and include the capsid protein (C), premembrane protein (prM) and envelope protein (E) [3]. The non-structural (NS) proteins are required for viral genome replication and include NS1, NS2A, NS2B, NS3, NS4A, NS4B and NS5 [3].

Eight distinct phylogenetic lineages have been proposed recently, of which lineages 1 and 2 are most widespread [4,5]. Lineage 1 strains have received a lot of attention as an emerging pathogen over the last two decades, and have been reported in Austria, Europe, North America and North Africa [6]. In contrast, lineage 2 strains have only recently been reported outside their historical geographic range of Madagascar and South Africa. Lineage 2 strains have reportedly been circulating in Hungary (2004) [7], Austria (2008) [8], Greece (2011) [9,10] and Italy (2012) [6,11].

The key mechanism for the generation of genetic diversity in WNV populations is mutation [12]. The WNV genome is subject to high mutation rates resulting from high error rates and the lack of proofreading ability of the RNA-dependent RNA polymerase [13]. Mutational change in structural and non-structural regions of the viral genome markedly influences cell tropism and host range [14]. The ability of a virus to cause infection depends on the recognition of cell surface receptors and intracellular host factors that permit virion multiplication and release [14]. Although genetic and phenotypic barriers provide the parameters associated with changes in cell tropism or host range, the evolutionary dynamics of viral subpopulations that accompany these changes are poorly understood [15].

This underlying population variation, termed viral quasispecies, is defined as a RNA population composed of a diverse mutant spectrum surrounding a master- or most dominant genotype that displays the highest fitness [16]. Large population sizes coupled with high mutation rates during viral replication result in increased genetic diversity within the mutant spectrum [17]. The heterogeneous viral strains present in viral quasispecies are classified into two components of variants, namely majority- and minority variants [18]. Interactions amongst components of the mutant spectrum determine the biological behaviour and phenotypic traits of a virus, and subsequently modulate the genetic diversity that is transmitted from infected hosts to susceptible hosts. The evolution of quasispecies is the result of evolutionary events that target the components of the mutant spectrum instead of the consesus genotype [18]. As a result, changes in quasispecies composition may take place without modification of the consensus genotype often interpreted as evolutionary stasis, potentially confounding identification of the genetic changes responsible for WNV emergence [17].

Drivers of WNV emergence, in particular genetic change, have been studied extensively in lineage 1 isolates after their introduction to the Americas. The majority of these studies, however, primarily focus on comparative analysis between genomic sequences generated from the consensus of all aligned reads of an isolate following whole genome sequencing [19]. Although NGS offers a cost effective, high-throughput method to generate hundreds to millions of short read genetic data in a single run, it has been utilised mostly for re-sequencing and comparative analyses of WNV [18]. Studies concerned with the maintenance of mutation-selection equilibria in WNV minority population variants throughout the time of emergence are, furthermore, greatly understated, especially so for lineage 2 WNV strains [18].

Advances in bioinformatic applications that reconstruct full-length haplotypes residing in viral quasispecies from ultra deep sequence data present a cost-effective and high-throughput method to study the presence and evolution of minority variants [18]. In this instance, each of the high number of sequence reads acquired from ultra deep sequencing is assumed to originate from an individual replicion, revealing meaningful underlying genetic variation [18]. In overcoming errors associated with PCR amplification, ultra deep sequencing, read filtering, read alignment, and the accurate detection of single nucleotide polymorphisms (SNP’s), haplotype reconstruction does not only provide insight into within-host viral evolution, but also epidemiological insight into dissecting possible transmission events [18].

This study presents the application of haplotype reconstruction from ultra deep sequence data to investigate the evolution of minority population variants of a historic South African lineage 2 WNV strain. The selective pressures that accompany changes in propagation environment was used as a simple model to firstly, study the influence of minority variant evolution on the consensus genome and secondly, to study the evolution and persistence of minority variants as individual replicons in the viral population. We present a cost-effective and high-throughput approach to the reconstruction of viral quasispecies that mitigates the main sources of possible sequence errors and biases. We confirm the results inferred from haplotype reconstruction by comparing single nucleotide polymorphism (SNP) profiles generated from the same sequence data in each instance where changes in propagation system were brought about. Results illustrate that minority variants contributing to cell-tropism persist in the viral population without contributing to changes in the consensus genome. Minority variants are shown, furthermore, to maintain a stable mutation-selection equilibrium under positive selection, particularly in the capsid gene region.

## Results and discussion

### Consensus genomes

The consensus genome of isolates propagated in mouse brain, BHK-21 cell cultures, and isolates that were switched from one propagation system to another displayed no genetic changes in the consensus genome sequence of isolate WNV 349/77 [Genbank: KM052152]. The lack of variation observed amongst consensus genome sequences of isolates WNV 1968 and WNV 349/77 is suggestive of a well-maintained mutation-selection equilibrium within the environment in which the respective isolates were propagated.

### SNP occurrence

**Figure 1:**
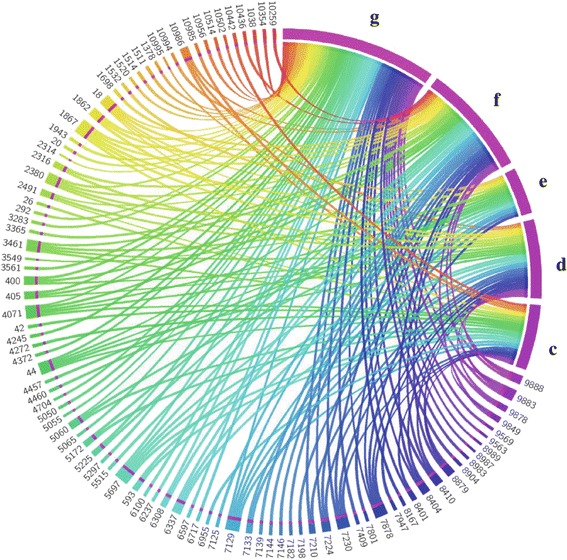
**Position and number of SNPs of WNV 349/77 isolates.** The genomes of isolates C3, D1, E1, F1, and G2 are each depicted by magenta bands. The numbers in the diagram depict the nucleotide position at which SNPs were detected. Genomes are connected to nucleotide positions where SNPs occur by means of a colour-coded ribbon. In the instance where a SNP is present in both genomes at the same nucleotide position, the nucleotide position is connected to both genomes by two similarly coloured ribbons.

A combined total of 98 unique SNPs were detected amongst all WNV 349/77 isolates (Figure 1). The number of SNPs that occurred in all five isolates comprised 7% of the combined total number of SNPs (Figure 1). The number of SNPs shared amongst four isolates, three isolates and two isolates was 4%, 11% and 9% respectively of the total combined number of SNPs each (Figure 1). The remaining 69% of the SNPs were unique to just one of the five isolates sequenced (Figure 1).

**Figure 2:**
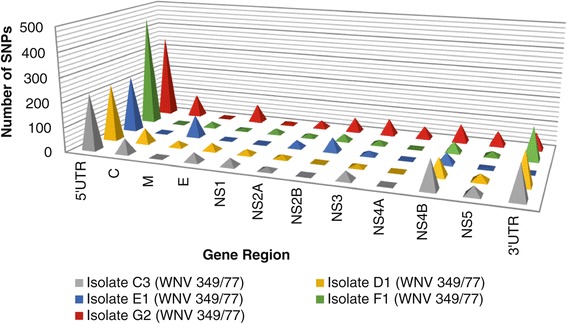
**Number of SNPs per gene region of WNV 349/77 isolates.**

In order to identify gene regions most influenced by changes in propagations system, the diversity of gene regions were compared based on the number of SNPs observed. The standardised number of SNPs per gene region for WNV 349/77 isolates was illustrated in Figure 2. The most variable region was identified as the 5′UTR region, followed by the 3′UTR region, the capsid gene region, the NS4B region, the membrane gene region, the envelope gene region, the NS3 region, the NS5 region, the NS4A region and lastly the NS1 and NS2A regions (Figure 2). The highest variation in number of SNPs between the respective isolates of WNV 349/77 was observed for the 5′UTR region, followed by the 3′UTR region, the capsid gene region, the membrane gene region, the envelope gene region, the NS4B region, the NS2A region, the NS5 region, the NS3 region, the NS4A region and lastly the NS1 and NS2A regions (Figure 2).

### SNP frequency

**Figure 3:**
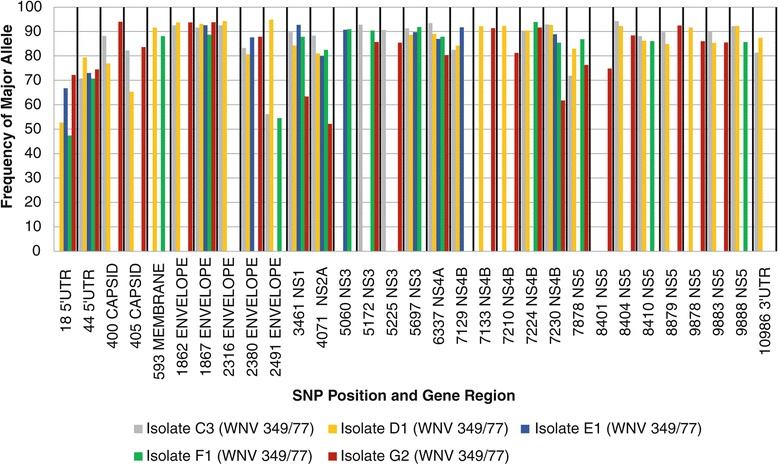
**Frequency of SNPs present in more than one WNV 349/77 isolate.**

The SNPs that occurred in all WNV 349/77 isolates and their respective frequencies based on the major allele are illustrated in Figure 3. The major allele at each SNP position remained identical between WNV 349/77 isolates, with the exception of SNPs at position 2316 and 2491. The SNP in position 2316 is situated in the envelope gene region. Whereas thymine is observed in position 2316 in the WNV 349/77 consensus genome, cytosine was observed in isolate C3 at a frequency of 92.5. This non-synonymous substitution resulted in an amino acid change from phenylalanine in the WNV 349/77 consensus genome to an arginine in isolate C3. This suggests that the substitution in position 2316 is under positive selection when the WNV 349/77 strain is passaged in mouse brain, and that the associated changes to the envelope protein confer elevated fitness in this propagation system.

Similarly, the SNP in position 2491 is situated in the envelope gene region. The WNV 349/77 consensus genome contains adenine in this position, whereas guanine is observed in isolate C3 and isolate D1 at a frequency of 56.1 and 94.9, respectively. This non-synomous substitution resulted in an amino acid change from isoleucine in the WNV 349/77 consensus genome to valine in isolates C3 and D1. Interestingly, adenine is observed at a frequency of 54.5 in isolate F1. Although the latter is agreement with the WNV 349/77 consensus genome, the frequency shows little deviation from that of guanine observed in isolate C3. Both isolate C3 and isolate F1 were propagated in mouse brain, but differed in the total number of passages with isolate C3 being passaged eight times and isolate F1 nine times. In contrast, isolate D1, was passaged 3 times in BHK-21 cell culture followed by one passage in mouse brain. When considering the passage history of all three isolates, the significance of the SNP at position 2491 becomes apparent. Firstly, the results suggest that quasispecies variants of WNV 349/77 that contain either adenine or guanine in position 2491 are maintained at a near equal frequency in the population. Secondly, the presence of adenine and the associated amino acid isoleucine in the envelope protein confers elevated fitness when WNV 349/77 is passaged in mouse brain, and the population equilibrium shifts towards adenine in position 2491 with the increase in passage number. In support of the latter, the high frequency at which guanine persists in isolate D1 when switching from three passages in BHK-21 cell culture to one passage in mouse brain suggests that the presence of guanine and the associated amino acid change to valine in the envelope protein confers elevated fitness in BHK-21 cell culture. The frequency of guanine declines sharply from 94.9 to 56.1 with increased number of passages in mouse brain as illustrated in isolate C3. This suggests that adenine in position 2491 is under positive selection when WNV 349/77 is passaged in mouse brain, whereas guanine is under positive selection when passaged in BHK-21 cell culture.

The envelope protein (E) is the most conserved of flavivirus structural proteins and is the major protein found on the virion surface [19]. The E protein mediates receptor binding and membrane fusion, and has two transmembrane segments that function as signal sequences for the transloaction of NS1 into the ER lumen [19]. Both the SNP’s at position 2316 and 2491 are situated in domain III of the E protein. As such, the increase in frequency of variants in position 2316 and 2491 reported after passage in mouse brain is suggestive of selective pressures brought about by the changes in receptor binding and membrane fusion between the central nervous system in mice and other propagation systems.

### Haplotype occurrence

**Figure 4:**
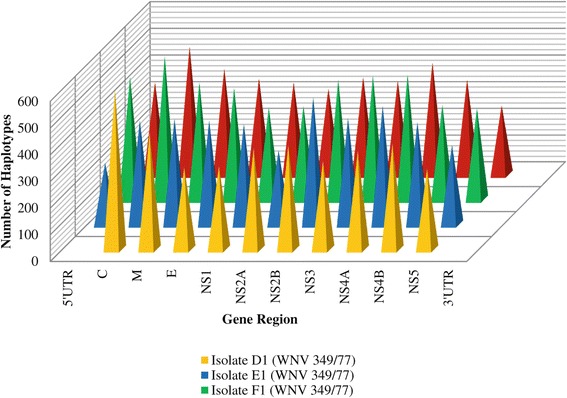
**Number of haplotypes per gene region of WNV 349/77 isolates.**

The haplotypes obtained consisted of full-length genomes aligned to the consensus genome of the isolate involved. The haplotypes obtained for isolate D1, isolate E1, isolate F1 and isolate G2 were compared (Figure 4). Based on variation in the number of haplotypes amongst gene regions, the most variable region of the WNV 349/77 genome was estimated as the 5′UTR region, followed by the capsid gene region, the NS3 region, the envelope gene region, the NS2B region, the NS2A and NS4A regions, the NS5 region, the membrane gene region, and the NS1 and NS4B regions (Figure 4). According to the variation in number of haplotypes between WNV 349/77 isolates within each gene region, the most variable regions were the 5′UTR region and the capsid gene region. These findings are in agreement with the gene regions found to be most variable according to SNP data.

### Haplotype frequency

**Figure 5:**
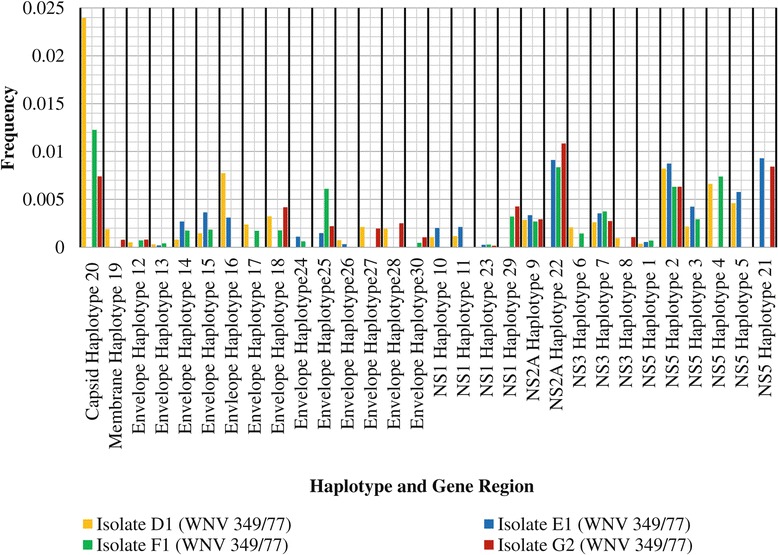
**Frequency of viable haplotypes present in each respective WNV 349/77 isolate.**

The frequencies of viable haplotypes that were present in more than one isolate were compared in order to study the influence of propagation system on the quasispecies composition of WNV 349/77. The shared haplotypes were renamed Haplotype 1 through to Haplotype 30 and grouped according to the gene region in which each respective haplotype displayed variation. The variation in haplotype frequency between isolates is illustrated in Figure 5.

Haplotypes that varied most in frequency between isolates were observed in the capsid gene region, followed by the envelope gene region and the NS2A region (Figure 5). The frequency of Haplotype 20 differed most between isolates, followed by Haplotype 25 and Haplotype 22 (Figure 5). For the purposes of this discussion, the emphasis will be placed on these three haplotypes.

Haplotype 25 contained variation in the NS2A region between genome position 367 and 3770. A pairwise distance of 0.0081 was observed between the genome sequence of Haplotype 25 and the WNV 349/77 consensus genome. Haplotype 25 was observed at a frequency of 0.01083 in isolate G2, 0.00912 in isolate E1 and 0.00835 in isolate F1 and was therefore most prevalent when WNV 349/77 was passaged continuously in BHK-21 cells.

Haplotype 22 differed in the envelope gene region from the consensus genome sequence of WNV 349/77. The pairwise distance between the genome sequence of Haplotype 22 and the WNV 249/77 consensus genome was estimated at 0.00667. Haplotype 22 contained variation in the envelope gene region between genome position 3672 and 3770. Haplotype 22 was observed at a frequency of 0.01083 in isolate G2, 0.00912 in isolate E1 and 0.00835 in isolate F1. Haplotype 22 was therefore most prevalent when WNV 349/77 was passaged continuously in BHK-21 cell culture, less prevalent when WNV 349/77 was subjected to a change in propagation system, and least prevalent when propagated continuously in mice. Similar to Haplotype 25, results suggest that Haplotype 22 is subject to selection when WVN 349/77 is propagated in BHK-21 cell culture.

The highest variation amongst WNV 349/77 isolates was observed in Haplotype 20. A pairwise distance of 0.00714 was observed between the genome sequence of Haplotype 20 and the WNV 349/77 genome sequence. With respect to the WNV 349/77 consensus genome, Haplotype 20 contained variation in the capsid gene region between genome position 221 and 318. Haplotype 20 was most prevalent when WNV 349/77 was subject to a change in propagation system from BHK-21 cell culture to mouse brain. An intermediate prevalence was observed when WNV 349/77 was continuously passaged in mouse brain, and a low prevalence when passaged continuously in BHK-21 cell culture. The magnitude in frequency variation observed between propagation in a constant environment and that of a change in environment suggests that Haplotype 20 provides a viable wild-type intermediate aiding in the process of adaptation. The latter indicates that the biological properties associated with the capsid protein that Haplotype 20 encodes is under positive selection during the transition of BHK-cell culture to mouse brain, without ultimately contributing to the capsid region consensus genome.

Both the 5′UTR and 3′UTR regions are highly variable amongst flavivirues [20,21]. The 3′UTR and 5′UTR contain common secondary structures necessary for genome replication (16). Similarly, the NS2A gene region is poorly conserved [22]. The NS2A protein interacts with replicase components of virus replication and coordinates the shift between RNA packaging and RNA replication [23]. The high variation observed in the 5′UTR region, the 3′UTR region and the NS2A region in comparison with other gene regions is therefore expected to occur.

In contrast, the high variation observed in the capsid protein gene region of WNV 349/77 is significant as the capsid protein forms an integral part in the assembly of infectious virions. The capsid protein facilitates membrane association and membrane protein (prM) translocation to the endoplasmic reticulum [24]. The assembly of RNA replication complexes, in turn, is known to occur on intracellular membranes [23]. As such, the increased variation in the capsid gene is suggestive of selection pressures brought about by differences in host cell type between propagation systems.

## Conclusions

The lack of variation observed amongst consensus genome sequences of WNV 349/77 isolates is suggestive of a well-maintained mutation-selection equilibrium within the environment in which respective isolates were propagated. This lack of variation in consensus nucleotide sequences is often interpreted as evolutionary stasis, and the underlying variation in the mutant spectrum that contributes to the consensus nucleotide sequence is neglected [15]. In this study, haplotype reconstruction from ultra-deep sequence data of a historic South African lineage 2 WNV strain revealed full-length haplotype genome sequences that depict this underlying variation. Variant frequency is shown to fluctuate mostly in the capsid gene region as positive selection persists to enable cell-tropism subsequent to propagation system changes. This is the first instance in which the quasispecies dynamics that ensure the continuity of WNV minority variants contributing to cell-tropism were shown to persist in the viral population solely from ultra deep sequence data. This study presents a cost-effective, high-throughput and accurate approach to full-length haplotype reconstruction of viral quasispecies by introducing bioinformatic measures of sequence error corrrection.

## Methods

### Ethics statement

This study was conducted with the approval of both the University of Pretoria’s Animal Ethics Committee (project number V006-13) and the Agricultural Research Council- Onderstepoort Veterinary Institute’s Animal Ethics Committee.

### Viruses

WNV 349/77 was isolated in South Africa in 1977 from a horse presenting with neurological symptoms. WNV 349/77 was received in lyophilised form after being passaged eight times intra-cerebrally in suckling mice; as well as three, five and seven times in baby hamster kidney (BHK-21) cell culture, respectively. For the purposes of this study, each of the four lyophilised isolates originating from the same viral stock of WNV 349/77 were passaged once more in either BHK-21 cell culture or intra-cerebrally in suckling mice and designated an additional isolate number according to passage history (Table 1).

**Table 1:** **Passage history of sequenced isolates**

**Strain**	**Isolate**	**Passage history**
WNV349/77	C3	Lyophilised as MB #8 → Sequenced
	D1	Lyophilised as BHK #3 → Passaged MB #1 → Sequenced
	E1	Lyophilised as BHK #5 → Passaged MB #1 → Sequenced
	F1	Lyophilised as MB #8 → Passaged MB #1 → Sequenced
	G2	Lyophilised as BHK #7 → Passaged BHK #1 → Sequenced

### Cell culture

BHK-21 cells were maintained in complete medium supplemented with 10% Fetal Calf Serum (FCS) (Invitrogen) until 90% confluency was reached. Complete medium was replaced with serum-free medium consisting of EMEM growth medium, 1% Penicillin/Streptomycin/Amphotericin B (Gibco), 1% L-glutamine (200 mM) (Gibco) and 1% non-essential amino acid (NEAA) (Gibco). Cells were transfected with filter sterilised virus originating from resuspended lyophilised material at a titre of 1.3 × 10^7^ TCID_50_ per mL. Infected cells were incubated at 37°C in 5% CO_2_ for 60 to 90 minutes. Medium containing virus was removed from cells and replaced by 2% complete medium. Infected cultures were incubated at 37°C in 5% CO_2_ for up to seven days. Virus was released from infected cell cultures demonstrating 50% cytopathic effect (CPE) by freeze-thawing. The cell culture material containing WNV was centrifuged at 15 000 rpm for 5 minutes and the supernatant was used for RNA extraction.

### Propagation in mice

The lyophilised material of each WNV isolate was resuspended in 1 mL phosphate buffered saline (PBS) solution. A further 1:10 dilution was prepared by the addition of 5 mL PBS to 500 μL of the original suspension. Suckling mice were each inoculated with a titre of 6.5 × 10^3^ TCID_50_ by intra-cerebral injection in the occipital region of the skull using a 28 gauge needle 6 mm in length. Mice were inspected three times daily for neurological- and behavioural symptoms of WNV infection during the seven day incubation period. At first sign of illness, mice were euthanized and virus was harvested from brain tissue. The brain tissue harvested during post-mortem was minced and used for RNA extraction.

### RNA extraction

Viral RNA was extracted according to the single-step RNA isolation method using TRIzol® reagent (Life Technologies). RNA quantity and purity was assessed by spectrophotometric measurements of the ratio of absorbance at 260 nm and 280 nm (NanoDrop). RNA was stored at −70°C.

### Ultra deep sequencing

The transcriptomes of WNV 349/77 isolates were amplified with the use of TransPlex Whole Transcriptome Amplification (WTA) kit (Sigma Aldrich) according to manufacturer instructions. The Illumina-compatible Nextera DNA Sample Prep Kit (EPICENTRE Biotechnologies) was used to prepare genomic cDNA libraries for sequencing according to manufacturer’s instructions. The DNA product recovered from tagmentation (simultaneous fragmentation and tagging of DNA with illumina adapters) was used as input for bridge PCR (bPCR) and cluster generation as per the standard Illumina protocol. Sequencing was performed using a HiScan system (Illumina) or MiSeq system (Illumina) at the ARC-Biotechnology Platform, Onderstepoort, Pretoria.

### Genome assembly

Illumina sequence reads were trimmed prior to genome assembly and mapping with the use of CLC Genomics Workbench v5.1.5 [25]. Reads were assembled *de novo* to optimise the paired read lengths of individual data sets. Trimming was repeated using data with read lengths conforming to a normally distributed range within the paired reads distance distribution (Table 2). Trimmed reads ranging between 79 bp and 82 bp in length were mapped to the complete genome sequence of lineage 2 WNV isolate SA 93/01 [GenBank: EF429198] to obtain a consensus sequence (Table 3). The consensus sequence was annotated accordingly.

**Table 2:** **Trimmed sequence read statistics**

**Strain**	**Isolate**	**Total read count**	**Trimmed read count**	**% Trimmed**	**Mean read length after trim (bp)**
WNV349/77	C3	1,646,236	1,653,756	99.36	79.4
	D1	2,054,204	2,051,074	99.85	80.7
	E1	1,620,826	1,618,204	99.84	82.5
	F1	1,972,894	1,969,863	99.85	82.1
	G2	1,929,256	1,926,236	99.84	81.4

**Table 3:** **Mapped sequence read statistics**

**Strain**	**Isolate**	**Total trimmed read count**	**Matched read count**	**Mean read length**	**Fraction reference coverage**	**Average coverage level**
WNV349/77	C3	1,625,850	628,670	90.99	1.00	4,125.35
	D1	2,051,074	693,868	82.43	1.00	5,478.51
	E1	1,618,204	963,103	84.36	1.00	6,923.01
	F1	1,966,832	175,646	82.68	1.00	1,202.94
	G2	1,923,216	371,000	82.71	1.00	2,543.41

### SNP detection

Single nucleotide polymorphisms (SNPs) were determined in CLC Genomics Workbench v5.1.5 [25] using the Neighborhood Quality Standard (NQS) algorithm [26]. The SNPs detected in sequence data of each isolate were annotated according to gene region. In order to identify gene regions most influenced by changes in propagation system, the diversity of gene regions were compared based on the number of SNPs observed. In each instance, the number of SNPs per gene region was scaled to the size of the genome to accommodate for the difference in size between gene regions. The standard deviation in the number of SNPs per gene region was calculated between isolates to identify the gene regions most influenced by changes in propagation system and passage number.

In order to study the influence of propagation system on quasispecies variation, the frequencies of SNPs that were shared amongst isolate D (WNV 249/77), isolate E (WNV 349/77), isolate F (WNV 249/77) and isolate G (WNV 349/77) were compared. In studying frequency changes of SNPs shared amongst isolates, all SNPs were considered regardless of codon changes resulting in premature stop codons. Due to the inability of the approach to discern the association between respective SNPs, quasispecies were reconstructed to obtain full-length haplotype sequence alignments.

### Quasispecies reconstruction

The full length haplotypes of WNV 349/77 isolates were reconstructed from ultra-deep sequence data in order to estimate the underlying genetic diversity that contributes to the quasispecies of each isolate. The sequence data of each isolate was aligned to its corresponding consensus genome as reference using the Burrows-Wheeler transform-based method [27] in Bowtie [28]. For every isolate, aligned reads were resampled to a total of approximately 50,000 reads to accommodate for the difference in the total number of mapped reads amongst isolates using SAMTools [29]. Each alignment was sorted and indexed to create a multiple sequence alignment (MSA) using SAMTools [29]. The MSA was subject to error correction and local haplotype construction by implementing a model-based probabilistic clustering algorithm [30] in ShoRAH [31]. The process was repeated for 5,000 iterations. The quality of the reconstructed haplotypes and corresponding frequencies were estimated in a Bayesian fashion by computing the posterior probability distribution of the aforementioned parameters [30]. Global analysis was performed on the corrected reads using a parsimony principle to compute the minimal set of haplotypes that explains the sequence data [32]. The frequencies of the haplotypes were estimated by maximum likelihood with the use of an Expectation Maximization (EM) algorithm [32]. Haplotypes with a posterior probability below 0.8 were discarded. The relative diversities of gene regions were compared between isolates based on the number of haplotypes recovered for each respective gene region. For each isolate, haplotypes with a posterior probability above 0.8 were annotated according to the gene region in which variation was observed. In order to identify gene regions most influenced by changes in propagation system, the diversity of gene regions were compared based on the number of haplotypes observed. In each instance, the number of haplotypes per gene region was scaled to the size of the genome to account for the size difference between gene regions. The standard deviation in the number of haplotypes per gene region was calculated between isolates to identify gene regions most influenced by changes in propagation system and passage number.

The frequencies of haplotypes that were present in more than one isolate were compared in order to study the influence of propagation system on the quasispecies composition of WNV 349/77. The sequences of haplotypes with a posterior probability above 0.8 were translated into protein sequences in MEGA5 [32] in order to identify and omit those containing nonsense mutations. The remaining haplotype sequences of each isolate were combined in a single nucleotide sequence alignment. The latter was implemented in DnaSP v5 [33] to identify haplotypes that were shared amongst isolates based on nucleotide identity. Due to the computational expenses involved in grouping full-length haplotype sequences, only haplotype sequences without stop codons in the WNV open reading frame were considered. The latter were compared based on the gene regions in which variation was observed.

### Availability of supporting data

The data set supporting the results of this article is included within the article (and its additional file).
